# Causes of mortality in patients after first‐ever stroke: A retrospective population‐based study

**DOI:** 10.1002/brb3.2294

**Published:** 2021-09-02

**Authors:** Arash Mosarrezaii, Mohammad Reza Amiri‐Nikpour, Sina Dindarian, Samerand Rahimzadeh, Sedra Mohammadi, Hozan Mohammadi

**Affiliations:** ^1^ Department of Neurology Urmia University of Medical Sciences Urmia Iran; ^2^ Student Research Committee Urmia University of Medical Sciences Urmia Iran; ^3^ Student Research Committee Tabriz University of Medical Sciences Tabriz Iran

**Keywords:** mortality, prevalence, risk factor, sex, stroke

## Abstract

**Background:**

Stroke is the third most common cause of death in developed countries and it is the most common cause of disability in the adult population of Iran. In this study, we aimed to evaluate the effects of age, sex, and other predisposing risk factors on mortality after stroke.

**Methods:**

We studied 1572 patients with first‐ever stroke during a 7‐year period from January 2008 to December 2014. Patients’ medical records including demographic information, past medical history, physical examination, and laboratory testing were reviewed. We analyzed the correlation of qualitative and quantitative variables with sex and mortality.

**Results:**

Of all patients, 252 (16%) died during the hospital stay and of the remaining 1320 patients, 453 (34.3%) died during the follow‐up period. There was no significant correlation between mortality and sex (*p* = .508). Descriptively, the number of women was higher in all age groups except in the age group 55–64 years. No significant correlation was observed between sex and age group (*p* = .748). We also observed a significant association between age group and mortality (*p* < .001). Hypertension is the most prevalent disease in both men and women. Higher levels of creatinine, urea, fasting blood sugar, neutrophils, cholesterol, and LDL significantly increase and higher levels of lymphocytes, platelets, RBCs, hemoglobin, and triglyceride significantly decrease the mortality.

**Conclusion:**

There are no sex differences in mortality after first‐ever stroke. Elderly patients need more support and attention due to greater stroke mortality. Complete blood count, lipid profile and blood levels of urea, creatinine, and fasting blood sugar may be useful in predicting mortality after first‐ever stroke.

## Graphical abstract

We aimed to evaluate the effects of age, sex, and other predisposing risk factors on mortality after stroke. We concluded that There are no sex differences in mortality after first‐ever stroke. Elderly patients need more support and attention due to greater stroke mortality. Complete blood count, lipid profile and blood levels of urea, creatinine, and fasting blood sugar may be useful in predicting mortality after first‐ever stroke.

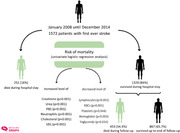

## 1 BACKGROUND

Stroke is the third most common cause of death after cardiovascular diseases and cancer in developed countries (Lopez et al., 2006; Murray & Lopez, 1997). In the United States, about 75,000 cases of cerebrovascular events occur annually. Almost 88% of these events are cerebral ischemic strokes of which, about 8–12% of them are fatal (Biller et al., 2005). Each year in Iran, 327 out of 100,000 people suffer from stroke, which is the most common cause of disability in the adult population of Iran (Salman et al., 2006). The non‐modifiable risk factors for stroke are age, sex, familial history of stroke, and race (Phillips, 2008; Terzis et al., 2003). Some modifiable risk factors for stroke include hypertension, smoking, peripheral vascular disease (PVD), carotid asymptomatic stenosis, atrial fibrillation, congestive heart failure (CHF), coronary artery disease (CAD), diabetes mellitus (DM), dyslipidemia, obesity, and inactivity (Greenberg et al., 2002; Simon et al., 2009). Risk factors for stroke are similar in women and men. However, at stroke onset, hypertension and atrial fibrillation (AF) are more prevalent in women, whereas heart disease, peripheral artery disease, diabetes, smoking, and alcohol consumption are the main risk factors in men (Reeves et al., 2008). Global stroke incidence and prevalence rates are respectively 33% and 41% higher in men than in women (Appelros et al., 2009). Although the greater prevalence of stroke in men is well known, recent evidence emphasizes the importance of stroke in women (Bousser, 1999). Stroke severity is greater in women than in men, (Lin et al., 1996; Palomeras et al., 2000) and the possibility of hospital discharge after acute stroke is sex‐related (Wyller et al., 1997).

In this retrospective study, we examined the effects of age, sex, and other important risk factors on the mortality rate of ischemic stroke. The results of this study may be helpful in improving preventive strategies and the in‐hospital management of stroke patients.

## 2 METHODS AND MATERIALS

In this retrospective population‐based study, 1572 patients were studied during a 7‐year period from January 2008 to December 2014 in Imam Khomeini Hospital, Urmia, Iran. Acute ischemic stroke (AIS) in patients was diagnosed by a neurologist using computed tomography (CT) scan or magnetic resonance imaging (MRI). Stroke was defined as sudden initiation of a focal (or global in case of coma) neurological disturbance of brain function lasting for more than 24 hours or leading to death apparently due to the vascular events (Aho et al., 1980). For collecting patients’ information, we reviewed available medical records in hospital files including demographic information, past medical history, physical examination, and laboratory testing. To determine the mortality of patients, their phone numbers were extracted from the files and recorded in the data collection form. Patients with incomplete data and those who died for any reason other than stroke such as infection, seizure, metabolic disorder, and any other disorder were not included in this study. Qualitative variables including sex, past history of hypertension (at least 2 blood pressure measurements >140/90 mmHg recorded before the stroke), PVD, coronary artery bypass grafting (CABG), CAD, DM (blood glucose levels >126 mg/dL for at least two measurements), AF, and myocardial infarction (MI), history of smoking, stroke subtype (embolic/thrombotic), ischemia location (anterior/posterior), and history of coronary angioplasty or stent placement, CHF, and dyslipidemia were analyzed in the patients. Embolism or thrombosis was established by a spiral CT or an MRI. Also, we analyzed quantitative variables encompassing age, levels of systolic blood pressure (SBP), diastolic blood pressure (DBP), creatinine, urea, blood sugar (BS), fasting blood sugar (FBS), white blood cells (WBCs), lymphocytes, neutrophils, platelets, hemoglobin, hematocrit, red blood cells (RBCs), cholesterol, triglyceride, high density lipoprotein (HDL) and low density lipoprotein (LDL), and follow‐up duration (month).

### 2.1 Statistical analysis

Quantitative variables are given as mean ± standard deviation (SD). Qualitative data are reported as numbers (percentages). We used a *t*‐test or Mann–Whitney test (if the normal distribution was difficult to assume) for continuous variables and the chi‐square test (if necessary, Fisher test) for categorical ones. The univariate logistic regression test was used to calculate the odds ratio for each variable in patients who survived and for those who died. Logistic regressions results were presented through the use of 95% confidence intervals (CIs). Statistical analyses were performed using SPSS version 20.0 (SPSS, Chicago, IL, USA). A *p*‐value of less than .05 was considered significant.

### 2.2 Ethical approval

All experiments were approved by the Ethics Committee, Urmia University of Medical Sciences, Urmia, Iran and were in accordance with the 1964 Helsinki declaration and its later revisions.

## 3 RESULTS

Out of 1572 patients, 744 (47.3%) were male and 828 (52.7%) were female. The mean age of the patients was 67.00 ±14.64. Five hundred and six (32.2%) patients had embolic and 1064 (67.8%) had a thrombotic stroke. All of the patients with thrombotic stroke underwent thrombolysis in the acute phase if there were no contraindications. Also, 953 (60.6%) patients had anterior circulation strokes (ACS) and 619 (39.4%) had posterior circulation strokes (PCS). Two hundred and fifty‐two (16%) patients died during the hospital stay and out of the remaining 1320 patients, 453 (34.3%) cases died during the follow‐up period. Of these 453 cases, 225 were male and 228 were female. There was no significant correlation between mortality and sex (*p* = .508). Also, the mean systolic and diastolic pressures were 137.00 ± 44.42 and 82.27 ± 13.67 mmHg, respectively. Furthermore, the mean follow‐up duration was 41.7 ± 21.6 months.

**FIGURE 1:**
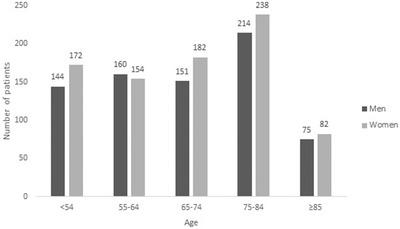
Sex and age distribution in patients with first‐ever ischemic stroke. No significant correlation was observed between sex and age group (*p* = .748)

**FIGURE 2:**
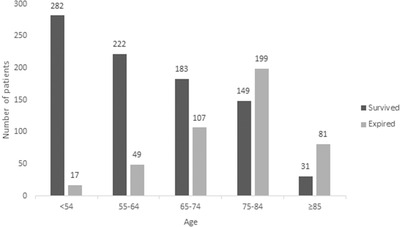
Mortality and age distribution in patients with first‐ever ischemic stroke. A significant correlation was observed between mortality and age group (*p* < .001)

Descriptively, Figure 1 demonstrates sex and age distribution and Figure 2 depicts mortality and age distribution in patients with first‐ever ischemic stroke. According to Figure 1, most of the patients in this study were 75–84 years old. The number of women was higher in all age groups except in the age group 55–64 years. Also, as seen in Figure 2, the highest and the lowest survival rates were observed in age groups <54 and ≥85 years, respectively. Additionally, among the patients younger than 75 years old, the number of survived patients was higher than the number of expired ones. But, among the patients aged greater than or equal to 75, the number of expired patients was higher. No significant correlation was observed between sex and age group (*p* = 0.748). We also observed a statistically significant association between age groups and mortality (*p* < .001).

Table 1 shows the prevalence of risk factors among the patients and the correlation of each variable with sex. As seen in the table, hypertension (*p* = .002), DM (*p* = .001), and smoking (*p* = .001) are the risk factors with a significant difference between men and women. In Table 2, we have presented the prevalence of risk factors according to mortality. All risk factors except AF (*p* = .354) were significantly different between the two groups. Tables 3 and 4 demonstrated the quantitative variables including age, blood pressure, and laboratory parameters according to sex and mortality, respectively. Age (*p* = .729), DBP (*p* = .282), BS (*p* = .082), WBC (*p* = .519) and neutrophil (*p* = .232) counts, triglyceride (*p* = .159) and LDL (*p* = .050) levels were not significantly different between men and women but all other parameters differed significantly between two groups. Also, all of the quantitative variables were different between both expired patients and survived patients except systolic (*p* = .256) and diastolic (*p* = .945) blood pressures, WBC count (.346), and HDL level (.092). Finally, the results of univariate logistic regression analysis demonstrated that higher levels of creatinine (*p* < .001), urea (*p* < .001), FBS (*p* < .001), neutrophils (*p* < .001), cholesterol (*p* < .001), and LDL (*p* < .001) significantly increase and higher levels of lymphocytes (*p* < .001), platelets (*p* = .044), RBCs (*p* < .001), hemoglobin (*p* = .003), and triglyceride (*p* = .034) significantly decrease the mortality in the patients. Table 5 shows the results of univariate logistic regression analysis for each quantitative variable in survived and expired groups.

**TABLE 1:** The prevalence of risk factors among the patients with first‐ever ischemic stroke and the correlation of each variable with sex

Risk factors	Variable definition	Men(744)	Women (828)	*p*‐Value	OR (95% CI)
Hypertension	Yes No	513 (69%) 231 (31%)	629 (76%) 199 (24%)	.002^*^	0.70 (0.56–0.57)
PVD	Yes No	119 (16%) 625 (84%)	145 (17.5%) 683 (82.5%)	.422	0.89 (0.68–1.170)
CABG	Yes No	39 (5.2%) 705 (94.8%)	32 (3.9%) 796 (96.1%)	.189	1.37 (0.85–2.20)
DM	Yes No	216 (29%) 528 (71%)	312 (37.7%) 516 (62.3%)	.001^*^	0.68 (0.54–0.83)
Dyslipidemia	Yes No	168 (22.6%) 576 (77.4%)	218 (26.3%) 610 (73.7%)	.085	0.81 (0.64–1.02)
CHF	Yes No	109 (14.7%) 635 (85.3%)	127 (15.3%) 701 (84.7)	.703	0.94 (0.71–1.25)
Coronary angioplasty or stent placement	Yes No	53 (7.1%) 691 (92.9%)	52 (6.3%) 776 (93.7%)	.504	1.14 (0.77–1.70)
CAD	Yes No	195 (26.2%) 549 (73.8%)	236 (28.5%) 592 (71.5%)	.309	0.89 (0.71–1.11)
MI	Yes No	63 (8.5%) 681 (91.5%)	52 (6.3%) 776 (93.7%)	.096	1.38 (1.80–2.94)
AF	Yes No	176 (23.7%) 568 (76.3%)	231 (27.9%) 597 (72.1%)	.55	0.80 (0.63–1)
Smoking	Yes No	225 (30.2%) 519 (69.8%)	131 (15.8%) 691 (84.2%)	.001^*^	2.37 (0.94–2.02)

Abbreviations: AF, atrial fibrillation; CABG, coronary artery bypass grafting; CAD, coronary artery disease; CHF, congestive heart failure; CI, confidence interval; DM, diabetes mellitus; MI, myocardial infarction; OR, odds ratio; PVD, peripheral vascular disease.

**TABLE 2:** The prevalence of risk factors among the patients with first‐ever ischemic stroke and the correlation of each variable with mortality

Risk factors	Variable definition	Survived(867)	Expired (453)	*p*‐Value	OR (95% CI)
Hypertension	Yes No	575 (66.3%) 292 (33.7%)	366 (80.8%) 87 (19.2%)	< .001^*^	2.13 (1.62–2.87)
PVD	Yes No	68 (7.8%) 799 (92.2%)	110 (24.3%) 343 (75.7%)	< .001^*^	3.76 (2.71–5.23)
CABG	Yes No	19 (2.2%) 848 (97.8%)	26 (5.7%) 427 (94.3%)	.001^*^	2.71 (1.48–4.96)
DM	Yes No	238 (27.5%) 629 (72.5%)	180 (39.7%) 273 (60.3%)	< .001^*^	1.74 (1.37–2.21)
Dyslipidemia	Yes No	165 (19%) 702 (81%)	132 (29.1%) 321 (70.9%)	< .001^*^	1.75 (1.34–2.27)
CHF	Yes No	58 (6.7%) 809 (93.3%)	105 (23.2%) 348 (76.8%)	< .001^*^	4.20 (2.98–5.93)
Coronary angioplasty or stent placement	Yes No	27 (3.1%) 840 (96.9%)	54 (11.9%) 399 (88.1%)	< .001^*^	4.21 (2.61–6.78)
CAD	Yes No	95 (11%) 772 (89%)	217 (47.9%) 236 (52.1%)	< .001^*^	7.47 (5.63–9.90)
MI	Yes No	35 (4%) 832 (96%)	54 (11.9%) 399 (88.1%)	< .001^*^	3.21 (2.06–5)
AF	Yes No	227 (26.2%) 640 (73.8%)	108 (23.8%) 345 (76.2%)	.354	0.88 (0.67–1.14)
Smoking	Yes No	173 (20%) 694 (80%)	119 (26.3%) 334 (73.7%)	.009^*^	1.42 (1.09–1.86)

Abbreviations: AF, atrial fibrillation; CABG, coronary artery bypass grafting; CAD, coronary artery disease; CHF, congestive heart failure; CI, confidence interval; DM, diabetes mellitus; MI, myocardial infarction; OR, odds ratio; PVD, peripheral vascular disease.

**TABLE 3:** The quantitative variables among the patients with first‐ever ischemic stroke and the correlation of each variable with sex

Variable	Sex	Mean ± SD	*p*‐Value
Age (years)	Male Female	67.23 ± 14.62 66.90 ± 14.67	.729
SBP (mmHg)	Male Female	134.73 ± 23.32 139.03 ± 57.02	.050^*^
DBP (mmHg)	Male Female	82.01 ± 13.63 82.5 ± 13.71	.282
Creatinine level (mg/dL)	Male Female	1.18 ± 0.65 1.08 ± 1.32	< .001^*^
Urea level (mg/dL)	Male Female	50.93 ± 36.55 45.76 ± 32.55	< .001^*^
BS (mg/dL)	Male Female	158.30 ± 89.83 163.63 ± 97.34	.082
FBS (mg/dL)	Male Female	129.13 ± 74.78 135.04 ± 72.96	.002^*^
WBC count (/μL)	Male Female	8744.55 ± 3752.03 8803.20 ± 6549.79	.519
Lymphocyte percentage (%)	Male Female	21.99 ± 13.74 23.22 ± 13.29	.026^*^
Neutrophil percentage (%)	Male Female	69.73 ± 15.61 69.04 ± 15.03	.232
Platelets (/μL)	Male Female	207.30 ± 79.44 228.67 ± 85.00	< .001^*^
RBC count (million/μL)	Male Female	4.61 ± 0.73 4.39 ± 0.67	< .001^*^
Hematocrit (%)	Male Female	40.14 ± 7.25 38.07 ± 9.61	< .001^*^
Hemoglobin (g/dL)	Male Female	12.96 ± 2.32 12.36 ± 2.66	< .001^*^
Cholesterol (mg/dL)	Male Female	187.42 ± 50.59 198.62 ± 57.99	.001^*^
Triglyceride (mg/dL)	Male Female	152.19 ± 72.16 157.21 ± 74.09	.159
HDL (mg/dL)	Male Female	45.13 ± 14.18 47.81 ± 23.45	< .001^*^
LDL (mg/dL)	Male Female	102.14 ± 39.18 107.27 ± 40.95	.050

Abbreviations: BS, blood sugar; DBP, diastolic blood pressure; dL, deciliter; FBS, fasting blood sugar; HDL, high‐density lipoprotein; LDL, low‐density lipoprotein, mg, milligram; mmHg, millimeter of mercury; RBC, red blood cell; SBP, systolic blood pressure; SD, standard deviation; WBC, white blood cell; μL, microliter.

**TABLE 4:** The quantitative variables among the patients with first‐ever ischemic stroke and the correlation of each variable with mortality

Variable	Mortality	Mean ± SD	*p*‐Value
Age (years)	Survived Expired	60.84 ± 14.20 75.22 ± 10.60	< .001^*^
SBP (mmHg)	Survived Expired	135.88 ± 23.37 139.38 ± 73.07	.256
DBP (mmHg)	Survived Expired	82.33 ± 12.59 82.26 ± 14.34	.945
Creatinine level (mg/dL)	Survived Expired	1.03 ± 0.53 1.16 ± 0.58	< .001^*^
Urea level (mg/dL)	Survived Expired	40.89 ± 23.22 52.88 ± 38.27	< .001^*^
BS (mg/dL)	Survived Expired	154.57 ± 94.58 163.01 ± 90.71	0.006^*^
FBS (mg/dL)	Survived Expired	121.59 ± 63.82 139.56 ± 77.41	< .001^*^
WBC count (/μL)	Survived Expired	8212.57 ± 3109.34 8363.65 ± 3152.63	.346
Lymphocyte percentage (%)	Survived Expired	25.06 ± 12.82 21.57 ± 13.31	< .001^*^
Neutrophil percentage (%)	Survived Expired	66.70 ± 14.73 70.16 ± 15.25	< .001^*^
Platelets (/μL)	Survived Expired	223.05 ± 80.65 213.80 ± 75.63	< .013^*^
RBC count (million/μL)	Survived Expired	4.59 ± 0.62 4.46 ± 0.74	< .001^*^
Hematocrit (%)	Survived Expired	39.48 ± 5.00 39.27 ± 12.50	< .036^*^
Hemoglobin (g/dL)	Survived Expired	12.83 ± 1.58 12.5 ± 2.53	< .001^*^
Cholesterol (mg/dL)	Survived Expired	183.27 ± 49.31 202.59 ± 57.41	< .001^*^
Triglyceride (mg/dL)	Survived Expired	157.88 ± 74.66 148.82 ± 71.01	.028^*^
HDL (mg/dL)	Survived Expired	46.33 ± 22.73 46.5 ± 13.83	.092
LDL (mg/dL)	Survived Expired	95.96 ± 32.33 114.18 ± 45.83	< .001^*^

Abbreviations: BS, blood sugar; DBP, diastolic blood pressure; dL, deciliter; FBS, fasting blood sugar; HDL, high‐density lipoprotein; LDL, low‐density lipoprotein; mg, milligram; mmHg, millimeter of mercury; RBC, red blood cell; SBP, systolic blood pressure; SD, standard deviation; WBC, white blood cell; μL, microliter.

**TABLE 5:** Results of univariate logistic regression analysis for each quantitative variable in survived and expired groups

Variable	*p*‐Value	OR (95% CI)
SBP level	.270	1.002 (0.999–1.005)
DBP level	.923	1.000 (0.991–1.008)
Creatinine level	< .001^*^	1.516 (1.202–1.912)
Urea level	< .001^*^	1.015 (1.010–1.019)
BS level	.123	1.001 (1.000–1.002)
FBS level	< .001^*^	1.004 (1.002–1.005)
WBC count	.405	1.000 (1.000–1.000)
Lymphocyte percentage	< .001^*^	0.978 (0.969–0.987)
Neutrophils percentage	< .001^*^	1.016 (1.008–1.024)
Platelet count	.044^*^	0.998 (0.997–1.000)
RBC count	.001^*^	0.739 (0.622–0.878)
Hematocrit	.675	0.997 (0.922–1.012)
Hemoglobin level	.003^*^	0.903 (0.845–0.966)
Cholesterol level	< .001^*^	1.007 (1.005–1.009)
Triglyceride level	.034^*^	0.998 (0.997–1.000)
HDL level	.880	1.000 (0.995–1.006)
LDL level	< .001^*^	1.013 (1.009–1.016)

Abbreviations: BS, blood sugar; CI, confidence interval; DBP, diastolic blood pressure; FBS, fasting blood sugar; HDL, high‐density lipoprotein; LDL, low‐density lipoprotein; OR, odds ratio; RBC, red blood cell; SBP, systolic blood pressure; WBC, white blood cell.

## 4 DISCUSSION

In the current population‐based study, we assessed the risk factors of mortality in patients after the first‐ever stroke. The most common age range of the first‐ever stroke was 75–84 years and only in the age group 55–64 years, the number of men was dominant. We did not assess the correlation between sex and incidence of first‐ever stroke because all of the patients in this retrospective study had stroke and we did not have control group to assess the correlation between sex and stroke incidence. Also, no significant correlation was found between age and sex. According to studies, sex differences highly depend on the patient's age in stroke incidence. In middle‐aged people, the rate of ischemic stroke in females begins to increase due to the onset of menopause and loss of sex hormones (Towfighi et al., 2007), and it stays high in elderly women (age >85 years) compared with elderly men (Bots et al., 2017). The population‐based study in Sweden reported a 60% lower incidence for stroke in women than in men at ages 55–64 years and a 50% higher incidence by the age of 75 years in women (Löfmark & Hammarström, 2007). Similarly, the Oxford Vascular Study found lower stroke incidence in women than in men aged 55–74 years, but the higher probability for women aged 85 years and older (Kissela et al., 2004). The WHO MONICA Project (18 European and Asian populations) surveyed 28‐day stroke mortality which was equivalent or higher in women than in men (Thorvaldsen et al., 1995). Sealy‐Jefferson et al. (2012) demonstrated that women are protected from stroke until almost 80 years of age in comparison with men and no sex differences in stroke risk were shown in their study. In the study of Rural Tianjin, China from 1992 to 2012 revealed that the incidence of stroke was lower in women than in men for all age groups, and the male/female incidence ratios decreased over time, especially in those aged ≥65 years (from 2.6 to 1.6 to 1.3). However, the incidence of stroke, annually, was greater in women than in men (8.0% versus 5.8%) from 1992 to 2012. Also, no significant sex difference in 30‐day mortality was observed in their study (Wang et al., 2014). Studies have demonstrated surprisingly controversial pieces of evidence about the correlation between stroke mortality and sex. Many studies have reported higher mortality (Di Carlo et al., 2003; Kelly‐Hayes et al., 2003; Niewada et al., 2005), while some have reported lower mortality in women (Asplund et al., 1988; Gillum et al., 1984; Reeves et al., 2008). In this study, we did not find any significant correlation between mortality and sex. We also observed a statistically significant association between age groups and mortality.

According to this study, risk factors vary depending on sex and mortality. Among predisposing risk factors, hypertension is the most prevalent in both men and women. Other most common risk factors in women are DM, CAD, AF, and dyslipidemia, respectively. Also in men, smoking, DM, CAD, and AF are respectively more prevalent after hypertension. The prevalence of hypertension, DM, and smoking was significantly different between men and women. In a review conducted by Reeves et al. (2008) they have also concluded that the prevalence of hypertension and AF in women with stroke are higher, whereas men experience a higher prevalence of heart disease, PVD, smoking, and alcohol use. Also, many other studies reported that women with stroke are older at onset (by an average of about 4 years), and are more likely to have AF and hypertension, whereas men with stroke are more likely to have a history of heart disease, MI, peripheral arterial disease, DM, and alcohol and tobacco use (Di Carlo et al., 2003; Holroyd‐Leduc et al., 2000; Kapral et al., 2005; Niewada et al., 2005; Roquer et al., 2003). Furthermore, Nationwide Danish Study (Andersen et al., 2010) demonstrated that before the age of 70–80 years, stroke risk factors were mostly more prevalent in men except for hypertension and AF, both being more prevalent in women. A recent review on sex differences in stroke also has demonstrated that women with stroke are more likely to have hypertension and AF, whereas, heart disease, MI, peripheral arterial disease, DM, and alcohol and tobacco use are more prevalent in men (Reeves et al., 2008). In the current study, the most common cause of death after the first‐ever stroke was hypertension. Among cardiac causes, only AF was not significantly related to mortality and this finding is in contrast with previous studies (Broderick et al., 1992; Ghatnekar & Glader, 2008; Kimura et al., 2005; Lamassa et al., 2001). In the study of Hannon et al. (2010) no differences were detected in the pre‐stroke modified Rankin scale (MRS) among AF and non‐AF patients with first‐ever ischemic stroke. However, those with AF had a greater acute neurological deficit compared to those with non‐AF stroke. Also, Wolf, et al. (Bordignon et al., 2012) surveyed 30‐day mortality rates in AF and non‐AF patients and did not find any differences (17% versus 19% respectively). They showed that recurrence in those with AF was not considerably frequent (25% versus 20%). The results of univariate logistic regression analysis in our study demonstrated that laboratory results reflecting impaired renal function such as creatinine and urea nitrogen are correlated with mortality. A study conducted by Krishna et al. (2009) have shown that patients with chronic renal disease have higher stroke risk. So, it may be concluded that chronic renal disease increases the risk of stroke and also the risk of mortality after stroke. Moreover, our results have shown that higher levels of cholesterol and LDL are associated with higher mortality but higher triglyceride levels decrease the risk of mortality. Also, we did not find any relationship between higher levels of HDL and lower risk of mortality. Studies have demonstrated that there is a correlation between lipid profile and mortality after stroke. The study of Zhao et al. (2018) has shown that higher levels of HDL are associated with decreased risk of stroke, but some studies have suggested that higher levels of total cholesterol, HDL, LDL, and non‐HDL cholesterol significantly decrease the incidence of stroke. They have also explained that higher levels of non‐HDL cholesterol can be a significant risk factor for the cardiac disease but not necessarily for stroke (Gordon et al., 1977; Sughrue et al., 2016). This can describe that cardiac disease could cause a higher mortality rate in patients with stroke. As most of the studies have evaluated the relationship between laboratory results and the risk of stroke, and since there are not many studies assessing the association of lab results with mortality after the first‐ever stroke, we recommend further studies in order to evaluate the correlation between laboratory findings and mortality after the first‐ever stroke. Also, follow‐up of the patients with regard to the mortality was performed using phone calls in most of the cases. Thus, we were not able to detect the causes of mortality after stroke during follow‐up period and this was one of the limitations of our study. Further studies especially cohort ones are recommended to be conducted in order to assess the causes of mortality in patients after first‐ever stroke. Also, parameters such as history of rehabilitation which could alter the patients’ prognosis were incomplete among the medical files, so we were unable to analyze them in the study. This was another limitation of the current study. Thus, we recommend considering these parameters in the future studies.

In conclusion, this study shows that there are no sex differences in mortality after first‐ever stroke. Also, we concluded that age groups determine important differences in patients suffering from first‐ever strokes and elderly patients need more support and attention because of greater stroke mortality. Furthermore, complete blood count, lipid profile, and blood levels of urea, creatinine, and FBS may be useful in predicting mortality after first‐ever stroke.

## CONFLICT OF INTEREST

The authors declare that they have no conflict of interest.

## FUNDING

This research did not receive any specific grant from any funding agency in the public, commercial or not‐for‐profit sector.

## AUTHOR CONTRIBUTIONS

All authors have read and approved the manuscript, and ensure that this is the case.

*Concept*: Arash Mosarrezaii and Mohammad Reza Amiri‐Nikpour. *Design*: Arash Mosarrezaii and Samerand Rahimzadeh. *Supervision*: Arash Mosarrezaii, and Mohammad Reza Amiri‐Nikpour. *Resources*: Samerand Rahimzadeh, Sina Dindarian, Sedra Mohammadi, and Hozan Mohammadi. *Materials*: Samerand Rahimzadeh, Sina Dindarian, Sedra Mohammadi, and Hozan Mohammadi. *Data collection and/or processing*: Samerand Rahimzadeh, Sina Dindarian, and Sedra Mohammadi. *Analysis and/or interpretation*: Arash Mosarrezaii, Samerand Rahimzadeh, Sina Dindarian, and Sedra Mohammadi. *Literature search*: Samerand Rahimzadeh, Sina Dindarian, Sedra Mohammadi, and Hozan Mohammadi. *Writing manuscript*: Sina Dindarian, Sedra Mohammadi, and Hozan Mohammadi. *Critical review*: Arash Mosarrezaii and Mohammad Reza Amiri‐Nikpour.

### PEER REVIEW

The peer review history for this article is available at https://publons.com/publon/10.1002/brb3.2294.

## Data Availability

The datasets used and/or analyzed during the current study are available from the corresponding author on reasonable request.
